# A Qualitative Exploration of the Built Environment as a Key Mechanism of Safety and Social Cohesion for Youth in High-Violence Communities

**DOI:** 10.1007/s11524-024-00861-z

**Published:** 2024-04-12

**Authors:** Lolita Moss, Kimberly Wu, Amber Tucker, Reanna Durbin-Matrone, Gabriella D. Roude, Samantha Francois, Lisa Richardson, Katherine P. Theall

**Affiliations:** 1 School of Public Health and Tropical Medicine, Tulane University, New Orleans, LA USA; 2 Violence Prevention Institute, Tulane University, New Orleans, LA USA; 3 Partners for Advancing Health Equity, School of Public Health and Tropical Medicine, Tulane University, New Orleans, LA USA; 4 Institute of Women and Ethnic Studies, New Orleans, LA USA; 5 School of Social Work, Tulane University, New Orleans, LA USA

**Keywords:** Built environment, Safety, Community violence, Social cohesion, Youth development

## Abstract

The characteristics of a neighborhood’s built environment may influence health-promoting behaviors, interactions between neighbors, and perceptions of safety. Although some research has reported on how youth in high-violence communities navigate danger, less work has investigated how these youth perceive the built environment, their desires for these spaces, and how these desires relate to their conceptions of safety and perceptions of other residents. To fill this gap, this study used focus group data from 51 youth ages 13–24 living in New Orleans, Louisiana. Four themes were developed using reflexive thematic analysis: community violence is distressing and disruptive, youth use and want to enjoy their neighborhood, systemic failure contributes to negative outcomes, and resources and cooperation create safety. This analysis indicates that young people desire to interact with the built environment despite the threat of community violence. They further identified built environment assets that facilitate socialization and recreation, such as local parks, and social assets in the form of cooperation and neighbor-led civic engagement initiatives. In addition, the youth participants demonstrated awareness of structural inequities that influence neighborhood health and violence-related outcomes. This study contributes to efforts to understand how youth with high levels of community violence exposure understand and interact with the built and social environments.

In addition to home and school, a neighborhood’s built environment (i.e., human-made or modified elements such as green spaces, population density, walkability, and esthetic qualities [1]) has important implications for youth development [2]. A growing body of research highlights the associations between the built environment and youth residents’ physical and mental health outcomes [3]. A recent systematic review reported that built environment features such as increased access to green space was related to fewer behavioral issues, lower stress, and greater well-being for youth [4]. These results suggest that the built environment may be one facet of the youth developmental ecosystem.

Just as favorable built environments may increase physical exercise and social cohesion, deteriorating built environments may shape the risk of violence. Scholars report that low-quality characteristics such as vacant and abandoned properties are associated with youth assault injury risk [5]. One study found that adolescent boys in low-income areas of Pittsburgh, Pennsylvania, had significantly lower odds of fighting if they lived in neighborhoods with higher walkability and fewer alcohol and tobacco stores [6]. Urban studies scholarship indicates that low-quality built environments negatively influence the social environment by generating mistrust, fear, and feelings of neglect, which in turn negatively affect social cohesion and residents’ mental health [7]. Higher social cohesion has been found to predict fewer depressive symptoms, greater levels of physical activity, and lower odds of delinquency among youth [8, 9]. Thus, the built environment may generate the physical conditions for violence, while reinforcing cognitive attitudes and symptomatology that buttress violence perpetration.

Built environment quality in areas of concentrated poverty across the United States reflects racial segregation and structural disinvestment [7]. Low-income neighborhoods are disproportionately affected by environmental toxicity, food deserts, unsafe housing conditions, deteriorating infrastructure, and violent crime [10]. New Orleans, Louisiana, occupies a unique context to further explore the interplay between violence, the built environment, and youth development. When the city was devastated by Hurricane Katrina, 25% of the city’s residents were living below the federal poverty line, nearly twice the national average [11]. In the aftermath of Katrina, properties in many lower-income neighborhoods, including the Lower Ninth Ward and New Orleans East, were abandoned [12]. Violent crime rates in the city spiked in the years following Katrina [13]. Today, predominantly Black neighborhoods in the New Orleans metro area have a lower life expectancy and higher poverty rates than their White counterparts [14].

Although the empirical scholarship is limited, research indicates that the built environment influences youth outcomes, in part, through perceptions of safety [15]. Qualitative research that investigates how youth in high-violence communities interact with their neighborhoods suggests that the threat of violence is salient and that youth adapt to the circumstances. Youth living in areas with frequent firearm violence have identified negative emotional effects of witnessing or hearing about such violence, including feelings of disempowerment and disengagement [16]. Other research has shown that youth have well-defined strategies for navigating the built environment to avoid danger and find public places to socialize, although these spaces are not always safe. Some examinations of youth perceptions’ about the built environment indicate that youth are unhappy with dilapidated and unkempt conditions [17, 18], yet little is known about what changes youth would like to see in the built environment, what assets they identify in the environment, how they conceive of safety, or their attitudes about fellow residents. These topics contribute to efforts to identify how the built environment influences youth development through cognitive processes of risk perception, identity development, and community orientation. The results of this research can be used to develop public health interventions to increase public safety and bolster healthy development for youth in underinvested communities.

Accordingly, the purpose of this study is to leverage the voices of youth to better understand their perceptions, usages, and desires for the built environment. To investigate these topics, this research used qualitative data from semi-structured focus groups of adolescents and young adults living in New Orleans, Louisiana. The following research questions guided our study:

1. What features of the neighborhood support youth safety?
2. What features of the neighborhood threaten youth safety?
3. How does exposure to community violence affect perceptions and use of the built environment?
4. How does exposure to community violence affect perceptions of the social environment?
5. How does community violence shape conceptions of safety?

## Method

### Design

This qualitative study utilized data collected for a parent study, the Healthy Neighborhoods Project (HNP), a cluster randomized controlled trial project that used a convergent, parallel design [19] to test vacant and abandoned property remediation as a mechanism to reduce community and household violence. The qualitative arm of HNP used focus groups to determine various stakeholders’ perspectives on violence, health, and the built environment in New Orleans. A total of 75 participants were interviewed across 11 focus groups and 23 key informant interviews. The current study used only youth focus group data (*N* = 51). This study was approved by the Tulane University Health Sciences Center Institutional Review Board.

### Recruitment

The youth participants were recruited using snowball sampling. Email announcements about the focus groups were shared with 72 community organizations beginning in October 2020 with a flier that included a link to register, study team contact information, and HNP’s website. Physical fliers were also posted around the city. Fliers for the online focus groups stated that participants would need a device with access to Zoom and a reliable internet connection. Prospective participants were screened using an online registration form that asked their age, parent/guardian contact information for those under 18, and availability. However, most participants did not complete this form and instead were interviewed while attending youth programming at community partner sites. Contact personnel at each site assisted with confirming eligibility; scheduling the date, time, and location for the focus groups; and obtaining parental consent.

### Participants

A total of 51 youth ages 13–24 years participated across five focus groups; the group sizes ranged from 1 to 14 participants. Fifty-one percent were girls. The majority reported their race as Black/African American (78.4%, *n* = 40), followed by 13.7% Hispanic/Latino (*n* = 7), 3.9% Native American (*n* = 2), and 3.9% White (*n* = 2). Fifty-six percent reported their age as 13–17 years old, and the remainder indicated that they were 18–24.

### Procedures

Focus groups took place from late October 2020 until January 2022. Each session was facilitated in after-school program settings, churches, or housing sites. The focus groups lasted approximately 60–90 minutes and were audio recorded. The focus groups were led by two research team members with qualitative data collection experience: one facilitated conversation and the other took notes. The research team members used a semi-structured interview guide that centered participants’ definitions of health and well-being, perceptions of the neighborhood and built environment, assessments of community violence and potential solutions, and the impact of COVID-19. The guide was piloted with three focus groups to help refine the questions.

All participants were provided a study consent form prior to participation that outlined the study team’s contact information, purpose, selection criteria, benefits, risks, confidentiality, incentives, and intent to record. The participants also received a copy of the interview questions and a demographics form prior to the interview. At each session, the consent forms were read aloud with time allocated to answer questions. Those who agreed to participate signed to indicate their agreement; minor participants obtained parental consent to participate and signed an assent form. Participants were compensated with a $25 gift card and received a mental health and anti-racism resource guide.

Some sessions were conducted over Zoom due to the COVID-19 pandemic. Changes to the protocol for virtual sessions included providing participants with a Zoom instruction guide in advance of the focus group, sharing guidelines to ensure privacy and comfort (e.g., the option for participants to turn off cameras), keeping microphones muted unless speaking, and the option to leave the group without penalty.

### Analysis

**Fig. 1:**
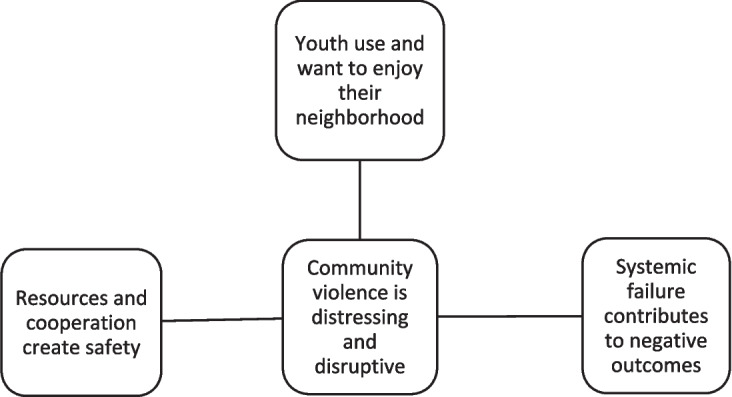
The thematic map depicts the four themes developed through Braun and Clarke’s reflexive thematic analysis six-phase process. The central theme, community violence is distressing and disruptive, unifies the three themes organized around it

The research team coded the data for latent and manifest content using a critical realist epistemological framework [20]. These data were analyzed following Braun and Clarke’s six-phase reflexive thematic analysis process [21]. The focus group audio recordings were initially transcribed using the transcript services Rev and Otter ai. In phase one, the transcripts were deidentified and verified for accuracy by two members of the research team, during which time potential themes related to the research questions were noted. Next, four research team members met regularly over several weeks to complete phases two through five. First, initial codes were generated and collected through Google Sheets. Second, a set of preliminary themes were developed that reflected patterns of data related to the research questions. Third, the themes were reviewed with the use of a thematic map as a visual aid to ensure the coherence of each theme and to verify that the map was an accurate reflection of the entire dataset. Lastly, the themes were defined and named, guided by some recoding of the entire data corpus, to ensure each theme’s internal homogeneity (i.e., coherence of the accompanying data extracts) and external heterogeneity (i.e., distinctiveness from the other themes). Four themes were developed at the end of this process: *community violence is distressing and disruptive*, *youth use and want to enjoy their neighborhood*, *systemic failure contributes to disparate outcomes*, and *resources and cooperation create safety.* See Fig. 1 for the finalized thematic map.

## Results

### Community Violence Is Distressing and Disruptive

The youth participants named community violence as a key safety concern. In addition to theft, carjackings, and fighting, firearm violence was emphasized across the focus groups. In one exchange, the participants discussed the frequency of hearing firearm violence:

Facilitator: So over here we also had some people say that they hear gun shots, and sometimes it feels like every day.

Participant 1: Not every day.

Participant 2: At least a couple times a week sometimes.

Participant 3: Like three times a week.

Participant 1: I ain’t hear no gun shots last week or the week before.

Participant 4: You must be deaf…There be a lot of gun shots.

One participant described the negative psychological effects of hearing gunshots, “it makes some people feel unsafe, mentally unstable, maybe [they] have panic attacks if there’s violence.” Other youth participants shared that frequent violence may induce paranoia, suicidality, or make residents feel “bad, because you can’t go nowhere really.”

Frequent among responses about the impact of such violence was how it disrupts the residents’ use of the built environment. Many stated that fear of victimization prevents them from moving freely about in the neighborhood: “I can’t walk to the corner store like how I used to. Because my mama scared that I might end up getting hurt, because they all be having shootouts every other day or every day, basically. And my mama’s scared to let me walk around all by myself.” Another participant noted that residents may avoid jogging or allowing their children to spend time at the playground as safety strategies. Thus, beyond individual psychological effects, violence disrupts the completion of errands, playground activity, and recreational use of public spaces.

### Youth Use and Want to Enjoy Their Neighborhood

The participants use the built environment for recreation and socializing, and they frequently related the two: “Well, we do have a park right by my house and in the neighborhood so that’s nice for people to take the stroll there and go and hang out. Everyone gets together there sometimes.” Other participants shared that they enjoy using a nearby football field for recreation and when a neighbor sell frozen treats known as “snowballs” to neighborhood youth. One person noted that young people are a key demographic who make use of the built environment: “I feel like we as kids, we as the youth, we come together to like, make our neighborhood a community. I feel like everybody else, they just go about they day, you know? They don’t go out their way; around here you kind of just fend for yourself…but us as the youth, we come together to be social within each other and stuff.”

Several participants shared that some amenities related to recreation and socializing are unavailable for use. For example, one person suggested fixing up a community garden: “I feel like a way that we could maintain improvements in our neighborhood is, since we have the little community garden, maybe we could start that up. And then, like I was saying, sustainability, we could stay consistent with the garden and make something out of it.” Another expressed frustration with their neighborhood’s broken swimming pool: “We need better. The people that come around here and fix stuff in your house, we need more because they don’t do nothing at all.” This theme underscores that youth with high exposure to community violence make needed adjustments to make use of the built environment and they demonstrate awareness that specific amenities are missed.

### Systemic Failure Contributes to Danger and Poor Health Outcomes

The youth related the causes of neighborhood hazards—including gun violence, blight, and faulty infrastructure—to structural failure and neglect. Most participants were Black youth who compared their communities to those with more White and wealthy residents. One participant suggested that power outages are inequitably addressed by the New Orleans utility company, Entergy: “They prioritize the West Bank and make sure that they power come on first. It’s true, y’all. And then they come to the East. I feel like they dedicate to the communities, like, who have a lot of money and stuff. They turn their power back on and all that type of stuff, and just give more to those type of communities.” Another youth stated that the lack of police presence leads to unsafe driving: “And uh, we don’t really have police officers to patrol the street where I live on, so people, they’ll drive real fast and you know, that’s how accidents happen and stuff like that.”

Across focus groups, there was a shared hypothesis that structural failure exacerbates violence and physical health outcomes. A participant related the location of a landfill to cancer rates: “The trash gets dumped under the houses and then, I think, the gases come up and then it might cause cancer. People, a lot of people have cancer there, probably because of all the trash there, we just figured out, people in the neighborhood just figured out about all the trash and all that.” There was a great deal of discussion about the lack of healthy food options in certain areas of New Orleans, and how it could influence health outcomes across generations:

“And I would kind of disagree with what you said, because, even if you feel like your neighborhood is safe, it’s the fact that they put McDonald’s and Burger King and um, Popeyes right into your neighborhood. They just feedin into the health crisis in the Black community. I know a lot of people with high cholesterol, um, and just passing down certain genes. Like if you, um…I forgot what gene it is, but it affects your kids and it’s in their blood too, because you eat a lot of, um, fast food and chemicals inside of the food. It’s not actual food, it’s just processed meat and everything.”

This theme captures how youth engaged in power analysis when discussing determinants of crime and poor health outcomes in predominantly Black, low-income areas of New Orleans.

### Resources and Cooperation Create Safety

The participants discussed tangible ways to increase feelings of safety via the built environment (i.e., fix flickering lights, pick up litter, and remove drug paraphernalia) and the need to ameliorate violence and delinquency with resources. For example, a young person noted that one neighborhood had few opportunities available for youth: “The only thing that’s up there that helps those kids, is like a youth program and that pays them to be inside, in there, because at least they know they’re there, but they can really use some more better resources.” Many participants shared that they felt much safer in school because of the security features and interpersonal connections:

Participant 1: Yeah, I feel safe inside and outside because there are teachers and security guards on both sides of my school, making sure that no one enters, no one exits.

Participant 2: I feel safe because we have a security guard and teachers and cameras that somebody watches.

Participant 3: I feel safe at school. But outside of school...we are in a bad neighborhood, so it’s not really that safe around.

Cooperation among neighbors and mutual understanding recurred as a key factor in neighborhood safety. One young person felt a sense of safety because of their neighbors:

“So in my neighborhood there’s like, majority of old people and it’s a mix also. And in our neighborhood they usually host meetings for our neighbors. And like, we talk to each other and since they’re older, they like to like, watch us. I had an old couple that watched me walk to the bus stop every morning for my safety, I guess.”

In the case of one participant who lived in an area of New Orleans that they described as gentrifying, they connected the influx of new neighbors to an increased sense of safety in the built environment:

My neighborhood has recently been getting a little bit better. Because of the... I mean, I don’t know exactly better, but there’s a lot of gentrification, and they’re building a bunch of new condos. And there used to be no condos in the Bywater, but now they’re building all these apartments. But so more people are moving in. So now when you’re walking around, there’s a lot more people. Because it used to be more scary, because you’d be walking around, and there only used to be one person. You know? But now you see a lot more tourists. You see just a lot more families. And that makes me feel a little bit better, because I used to not be able to walk around by myself. But now it’s easier to get out of the house.

Community organizing and volunteerism were referenced as one avenue to increase cooperation and to address issues:

“I think organizing groups of the youth or maybe older adults or anyone really that wants to help out to maintain those said trash cans in the areas that are needed, like in areas that you wouldn’t really see these being placed at and just keeping them as a reminder that this is a responsibility for them. It helps the community, and having a large group of people that are willing to do this without pay or anything, with just the realization that they are helping the community.”

There was frequent openness to community-based solutions and leaning on cooperation as a viable path to increase neighborhood safety.

## Discussion

This qualitative study investigated the ways that a sample of youth living in high-violence areas of New Orleans, Louisiana, perceive and interact with the built environment, their conceptions of safety, and how violence exposure influences perceptions of fellow community members. Four key themes were developed through reflexive thematic analysis of focus group data: *youth use and want to enjoy their neighborhood*, *community violence is distressing and disruptive*, *systemic failure contributes to negative outcomes*, and *resources and cooperation create safety*. This research contributes to the scant literature that has identified developmental assets in high-violence communities, how youth conceptualize safety, and their critical reflection of the influence of structural entities on the built and social environs.

This study confirms previous research that youth in high-violence neighborhoods find ways to engage the built environment and adapt to potential danger [22]. The finding that youth in high-violence areas desire improvements to the built environment for recreation and socializing (e.g., the community pool), however, has not been investigated in-depth in the empirical literature. Similarly, little work has documented assets in high-violence neighborhoods that may support youth development or safety. The youth participants identified built environment assets such as spaces for leisure time. The focus group data indicate additional assets in the social environment; some participants shared that neighbors who look out for them or raise awareness about neighborhood-related issues increase their level of safety and comfort. It is relevant to note, too, that time spent with friends was named as an important neighborhood benefit. One participant stated that it is the youth who “come together to, like, make our neighborhood a community.” Other qualitative research has reported the importance of social opportunities for youth in high-violence neighborhoods [23, 24], and developmental perspectives indicate that high level of socializing with peers is a normative dimension of adolescence [25]. However, much research examines the negative influence of peers [26]. Given that youth in high-violence areas may view friends as one of few assets in their neighborhoods, perhaps public health-related interventions could leverage these relationships, as some interventions have positive effects when using peer models [27]. Watchful neighbors who look out for youth could also be engaged in place of parents who may not have the time or ability to engage in interventions.

This analysis suggests that the youth participants understand that perceived threats in the built and social environment can affect mental health and threaten social cohesion [28]. Across focus groups, the youth hypothesized that adult residents may be less open to socializing due to safety-related stressors, which are exacerbated by vacant properties and disrepair in the built environment. Scholars have argued that perceptions of the built environment influence perceptions of neighborhood residents, and hazards in the built environment may contribute to social isolation [29]. Although urban studies scholarship has documented that many youth fear neighborhood changes brought on by gentrification [30], some participants celebrated new neighbors brought into the community by gentrification because the influx of people communicates safety that deserted spaces do not. These findings have important public health implications. Because youth are compelled to congregate outdoors for leisure, one strategy to improve high-violence areas may be updating broken amenities, cleaning abandoned properties, and other “beautification” efforts. As has been recommended elsewhere [17], youth could be involved in such interventions, as such involvement has been associated with a stronger sense of community and individual investment in the community [31]. Future research should seek to understand if youth community members experience psychosocial benefits from built environment improvements brought on by gentrification. Conversely, scholars must document potential threats to social cohesion catalyzed by new neighbors who may engage in, for example, citizen policing [30].

Little qualitative work has studied youth residents’ awareness of systemic factors that influence the built environment and social inequality. The theme *systemic failure contributes to negatives outcomes* captures how participants fluidly named macro-level sources of health inequity. Their theories about the root causes of juvenile delinquency and disproportionate fast food access demonstrate some level of *critical consciousness*, a framework developed by Brazilian educator Paulo Freire that refers to a process by which marginalized youth learn about structural inequality and are moved to social action [32]. Similarly, in the theme *resources and cooperation create safety*, youth emphasized the lack of resources apportioned to the residents of their respective communities across New Orleans. In addition, the mechanisms of their safety recommendations were largely interpersonal (i.e., community organizing, volunteerism, and more youth programs). This perspective adheres to critical consciousness, which underscores the power of communities to realize social change [33]. The sources of these youths’ critical reflection is unclear and may be related to understanding systemic racism via racial socialization [34]. Given that recent scholarship suggests critical consciousness may be an appropriate strategy to address health inequity [27], public health and urban studies scholars can investigate levels of critical consciousness as a potential buffer against the negative developmental outcomes associated with residence in high-violence areas.

This research has some limitations that should be acknowledged. First, although our sample size is appropriate for our methodological approach, these data are not necessarily generalizable. Our novel findings must be validated with further empirical research to best inform policy and practice. Second, participants were recruited through community-based organizations and thus represent a convenience sample. Third, the focus groups were mixed-gender and accommodated youth across a wide age range. Therefore, it is possible that gendered or age-specific perceptions of safety, the built environment, and community members were obscured in this analysis. Despite these limitations, these findings represent a meaningful contribution to scholarly work on adolescents living in high-violence, disinvested communities in New Orleans.

## Conclusion

Scant empirical work advances our understanding of how the built environment influences youth and young adults’ desires and perceptions of their neighborhoods, their conceptions of safety, or their understanding of structural forces that overdetermine negative outcomes. This research attempts to fill that gap in knowledge with an analysis of qualitative focus group data from youth living in areas of New Orleans with high levels of community violence. These results contribute to scholarship about the ways in which the built environment contributes to youth perceptions of neighborhood assets, structural influences of crime, and the social environment.

## Data Availability

The raw data analyzed for this study are not publicly available to preserve participant privacy. Deidentified data may be made available upon request.
